# Body mass index in relation to truncal asymmetry of healthy adolescents, a physiopathogenetic concept in common with idiopathic scoliosis: summary of an electronic focus group debate of the IBSE

**DOI:** 10.1186/1748-7161-8-10

**Published:** 2013-06-25

**Authors:** Theodoros B Grivas, Geoffrey R Burwell, Peter H Dangerfield

**Affiliations:** 1 Department of Trauma and Orthopedics, “Tzanio” General Hospital, Tzani and Afendouli 1st, Piraeus 18536, Greece; 2 Centre for Spinal Studies and Surgery, Nottingham University Hospitals Trust, Queen’s Medical Centre Campus, Derby Road, Nottingham NG7 2UH, UK; 3 University of Liverpool, Ashton Street, L69 3GE, Liverpool, UK; 4 Staffordshire University, Leek Road, Stoke-on-Trent ST4 2DF, UK; 5 Royal Liverpool Children’s Hospital, Eaton Road, Liverpool L12 2AP, UK

## Abstract

There is no generally accepted scientific theory for the cause of adolescent idiopathic scoliosis (AIS). As part of its mission to widen understanding of scoliosis etiology, the International Federated Body on Scoliosis Etiology (IBSE).introduced the electronic focus group (EFG) as a means of increasing debate on knowledge of important topics. This has been designated as an on-line Delphi discussion. The text for this debate was written by Dr TB Grivas. It is based on published research from Athens, Greece evaluating schoolchildren age 11–17 years for the relation of body mass index (BMI) to each of truncal asymmetry (TA) and menarcheal status. Girls with relatively lower BMI were found to have a significant excess of severe TAs and significantly later menarche confirming the well-known relation of BMI to menarche. Together with other evidence linking nutritional status to skeletal growth, the observations suggest energy balance via the hypothalamus is related to trunk asymmetry. As with a recent speculative hypothesis for the pathogenesis of AIS in girls, Grivas et al. suggest that the severe TAs involve a genetically-determined selectively increased sensitivity (up-regulation) of the hypothalamus to circulating leptin with asymmetry as an adverse response to stress (hormesis). The TA is expressed bilaterally via the sympathetic nervous system to produce left-right asymmetry in ribs and/or vertebrae leading to severe TAs when beyond the capacity of postural mechanisms of the somatic nervous system to control the shape distortion in the trunk. This EFG discusses the findings and interpretations of the paper by Grivas and colleagues as research at the borderland between the genesis of TA (physiogenesis) and AIS (pathogenesis). It is suggested that TAs, here regarded in common with AIS, result from the combination of secondary sexual development affecting body composition, adolescent skeletal growth velocity, and an asymmetry process. The possible involvement of epigenetic factors is not considered.

## Background

Most experts agree that the cause of adolescent idiopathic scoliosis (AIS) is multifactorial with no generally accepted theory of pathogenesis [1,2]. AIS may not be one disease but several types all with different initiating mechanistic processes going wrong and common factors contributing to progression [3]. This overall view reflects shortcomings in our understanding of the complex biological and biomechanical multifactorial processes, possibly interacting [4], involved in AIS pathogenesis which need innovative thinking [5], to which may be added new findings not explained by prevailing theories. In the latter connection, a novel speculative hypothesis for AIS pathogenesis was formulated recently from comparing skeletal growth and maturation in girls with higher and lower body mass index (BMI) subsets relative to median values; This double neuro-osseous theory involves the somatic and autonomic nervous systems in AIS pathogenesis [6].

Hitherto, the focus by Grivas and colleagues for the pathogenesis of idiopathic scoliosis concerned rib-verrtebra angle asymmetry, growth, the somatic central nervous system, and gait (dinner-plate flagpole concept) [7,8]. As the basis for the present paper, Grivas and colleagues [9] studied healthy adolescents age 11–17 years (girls 2930, boys 2817). Using BMI they found *lower BMI subsets* to be associated with an excess of severe truncal asymmetry (TA)*.* In their research, TA was assessed during screening for scoliosis in a sitting, rather than a standing forward bending (FB) position; this was adopted because sitting FB readings express TAs free from the effects on TA of any leg-length inequality in the standing FB position. TA was measured as angle of trunk inclinations (ATIs) across the back in each of three regions, thoracic, thoracolumbar and lumbar, with abnormality defined as 2 standard deviations or more.

Grivas et al. [9] found:

•no sex difference in TAs for any of the three spinal regions, thoracic, thoracolumbar and lumbar;

•severe TAs relative frequency was significantly higher in girls than boys for each of the right thoracic and thoracolumbar regions; and

•excess of abnormally increased TAs, and later menarche were each associated statistically with. relatively lower BMIs.

In interpreting their findings, Grivas et al. [9] speculatively applied to TAs the autonomic component of the double neuro-osseous pathogenetic theory for girls with AIS [6], Grivas et al. suggested that severe TAs involve a *genetically-determined selectively* increased hypothalamic sensitivity to leptin with asymmetry as an adverse hormetic response (hormesis). TAs are viewed putatively as being expressed bilaterally via the sympathetic nervous system to produce left-right asymmetry in ribs and/or vertebrae leading to severe TA when beyond the capacity of postural mechanisms of the somatic nervous system to control the shape distortion of the trunk. Possible epigenetic mechanisms [10] were not considered.

This EFG discusses the findings and interpretations of the paper by Grivas and colleagues [9] as research at the borderland between the genesis of TA (physiogenesis) and AIS (pathogenesis). Grivas et al. suggest here that the phenotypic sexual dimorphism of normal TAs is related to the onset and timing of puberty including the combination of three components, namely: 1) s*econdary sexual development affecting body composition, with g*irls depositing more white adipose tissue than boys, affecting circulating leptin levels; 2) *adolescent skeletal growth velocity* and 3) a putative *asymmetry process* as suggested recently for AIS girls [11,12]. Both 1) and 2) and, in some AIS girls, possibly 3) are initiated and controlled by hypothalamic and local mechanisms [11-13]. This physiopathogenetic concept, in common with idiopathic scoliosis does not exclude the possibility of somatic nervous system mechanisms contributing to the causation of TAs in some healthy subjects as suggested for the trunk deformity of some AIS girls [6].

### Statement by Dr Grivas

In a recent publication [9], the relation between truncal asymmetry (TA), body mass index (BMI) and menarcheal status was evaluated in healthy adolescents age 11–17 years (boys 2817, girls 2930).

(1) Each child was assigned a value of “lower” or “higher” BMI relative to median values constructed for each age group, gender, position and spinal region.

(2) The sitting FB position is thought to express intrinsic TA free from extrinsically-induced effects of any leg-length inequality. The analyses of the findings for the pathogenetic interpretation utilise these data.

(3) In the sitting FB position for severe TA: a) there is no sex difference in TAs for any of the three spinal regions, thoracic, thoracolumbar and lumbar; b) an excess of right relative to left ATIs outside 2 SDs in both boys (p = 0.020) and girls (p = 0.009) p values; c) the relative frequency of severe TAs is significantly higher in girls than boys for each of the right thoracic and thoracolumbar regions; and d) after correcting for age, lower BMIs are associated with more severe TAs in boys and girls.

(4) Mean estimated age at menarche was significantly later in the lower BMI subset, than in the higher BMI subset (mean and SD respectively 12.10 ± 1.21 years, and 11.61 ± 1.26 years, p < 0.001).

(5) The girls with relatively lower BMI are associated with significantly later menarche, and a significant excess of severe TAs. These observations together with other evidence [6] suggest a relation of trunk asymmetry to energy balance via the hypothalamus.

(6) As with a recent hypothesis for the pathogenesis of AIS in girls [6], it is suggested that severe TAs are caused by a genetically-determined selectively increased sensitivity (up-regulation, i.e. increased sensitivity) of the hypothalamus to circulating leptin with asymmetry as an adverse response to stress (hormesis)(LHS concept). This hypothalamic functional asymmetry is expressed via the sympathetic nervous system bilaterally to produce left-right asymmetry in ribs and/or vertebrae leading to severe TAs, when beyond the capacity of postural mechanisms of the somatic nervous system to control the shape distortion in the trunk [6].

(7) It is suggested that the majority of girls and boys with TA who do not progress to AIS [14], may have less severe involvement of their autonomic and somatic nervous systems. They may also lack the hormonal changes [6] and osteopenia [15] of preoperative girls with AIS [6], both of which may contribute to the curve severity and the biomechanical spinal growth modulation of progressive AIS [3].

### Comments, questions and responses

#### Trunk asymmetry

##### Comment no1

I have observed trunk asymmetry (TA) in normal children. When it occurs it is connected with the “Syndrome of Contractures” described by Professor Mau [16].

Response

Yes, there is trunk asymmetry (TA) in normal children – the “constitutional trunk asymmetry” [14,17,18] which is not necessarily connected with, or due to, the “Syndrome of Contractures [16]”.

#### Other bilateral asymmetries

##### Comment no2

Dr. Grivas’ cohort is very impressive. I assume by trunk asymmetry he is referring to the spinal column and ribcage and does not include the asymmetric shoulder line or oblique pelvis.

Response.

Yes, our research refers to trunk asymmetry (TA) related to the spinal column and ribcage, not to the shoulder line, or oblique pelvis.

In many patients with AIS there is also an asymmetric shoulder line and/or oblique pelvis which were not recorded in our data collection.

Some skeletal imaging studies of the trunk including asymmetries are:

•rib-vertebra angle (RVA) asymmetry [7,19,20],

•thoracic ratios [21],

•convex/concave rib-hump index (‘double rib contour sign [22,23];

•vertebral sagittal profile [24-26],

•radiographic axial vertebral rotation [27-29],

•ultrasound axial vertebral and rib rotations [30], and

•ultrasound spine-rib rotation difference (SRRD) [31].

In extra-spinal sites (other than the CNS), widespread bilateral asymmetries have been detected in AIS subjects (see Appendix):

•*in the trunk -* breasts [32,33], ribs [34-39], chest wall blood supply [40,41], and iliac height [42,43];

•*in upper limbs,* upper arms [11,12,44] and forearm-with-hands [45];

•*in lower limbs,* femoral neck-shaft angles [46], femoral anteversion [47,48], femoro-tibial correlations [49,50], ilio-femoral length [51], tibial length [43,52] but not tibial torsion [48,53],

•*in the head*, skull vault [54], face [55], orthodontic anomalies [56,57] and vestibular apparatus [58].

Widespread bilateral skeletal asymmetries characterise the phenotype of idiopathic scoliosis. Surface trunk asymmetry, upper arm length asymmetry and iliac height asymmetry each correlate significantly with adjacent spinal curve severity suggesting a common pathogenesis.

#### Scoliometer readings

##### Comment no3

I have problems with the Scoliometer on two counts:

(a) it is not accurate, and

(b) we are bi-pedal and do not live on all fours.

If scoliosis surgeons only used radiographs of the forward bending test, i.e. rib horizon, I doubt if many would be keen for surgery. As Bunnell [59] and others have shown, rib asymmetry does not always equate with vertebral column asymmetry.

Response.

The accuracy (intra- and inter-reliability) of the Scoliometer has been reported, Grivas et al. [60] (Table 1).

**Table 1:** **The accuracy (intra- and inter-reliability) of the Scoliometer [**[60]**] reliability study of recorded asymmetry**

	**Intra-observer error**	**Inter-observer error**
Mid thoracic (T4-T8)	1.8	2.6
Thoracolumbar (T12-L1)	3.2	3.3
Lumbar (L2-L5)	4.0	4.3

Murrell et al. [61] reported on Scoliometer reliability. They noted that there was almost perfect agreement between the thoracic Scoliometer measurements in degrees with an intra-reader error of 1.2 degrees; a similar high agreement was observed in lumbar Scoliometer measurements in degrees with an intra-reader error of 1.6 degrees. They highlighted that the Scoliometer can be used reliably by a single trained observer to measure trunk rotation; and that their current practice uses the Scoliometer in every physical examination of patients being screened for, or with, scoliosis.

The question for clinical practice is: What exactly does the Scoliometer assess? It assesses surface ribcage asymmetry, and not spinal column deformity, traditionally assessed using radiographs (Cobb angle). These measurements assess two quite different morphologies. In a number of publications, the authors have evaluated the correlation of these measurements with a view to reducing radiation exposure in clinical assessment [22,23,62,63].

Amendt et al. [64] reported that Scoliometer measurements made by two raters on 65 persons with idiopathic scoliosis correlated with radiographic agreement of both vertebral (pedicle) rotation and lateral curvature (Cobb method). Correlations with pedicle rotation ranged from 0.32 to 0.46 and with Cobb angle from 0.46 to 0.54. Frequency analysis revealed relatively good specificity, sensitivity, and predictive capability of the Scoliometer. Intra-rater and inter-rater reliability coefficients were high (r = 0.86-0.97). These results indicate good measurement reproducibility. The less than optimal between method correlation coefficients suggest that the validity of Scoliometer measurements is not sufficient to use this method alone for determining patient diagnosis and management. But based on positive-frequency analysis the use of this instrument as a screening device would be appropriate. These findings can be accepted especially if we consider some new knowledge based on the recent published research on the correlation of surface (truncal) and axial (spinal) deformity by Grivas et al. [23].

Grivas et al. [23] reported that in girls with idiopathic scoliosis, growth has a significant effect in the correlation between the thoracic and spinal deformity In younger children, the concordance of surface and spinal deformity is weak and becomes stronger as the children grow. Therefore, in younger children with surface/trunk asymmetry, the prediction of the spinal deformity alone from the surface topography is inaccurate. This knowledge relating to age should be taken into consideration when assessing spinal deformity from surface measurements, and correlating surface and radiological readings.

Huang et al. [62] reported on the effectiveness of this instrument and studied the correlation of Scoliometer with radiographic readings. They concluded that the value of the Scoliometer in school scoliosis screening still needs further evaluation. This statement in our opinion underestimates the value of the Scoliometer for screening asymmetry and is not acceptable. The age range of screened children by Huang et al. was 12 – 14 years old. As we discuss below in this age range of screened children, the correlation of surface and radiographical deformity is not statistically significant, therefore the author’s findings were expected and predicted.

We reported [22] that in children younger than 14 years with a double rib contour sign (DRCS) and rib hump (rib index >1), the correlation of surface (Rib index) and radiographical (Cobb angle) deformity is not statistically significant, which is the case in children older than the age of 14. Analysing the above statement it is useful to say that all lateral spinal radiographs in idiopathic scoliotics show a double rib contour sign (DRCS) of the rib cage, a radiographic expression of the rib hump. The outline of the convex ribs overlies the contour of the concave ribs. The DRCS, results primarily from rib deformation and secondarily to vertebral rotation, because DRCS could be present in straight spines with no vertebral rotation. In all our school-screening referrals having ATIs >7°, the thorax deformity in terms of the DRCS/hump has already developed; 70% of these children were scoliotics. The rest had a curvature of less than 9° of Cobb angle (10%), or they were children with straight spines (20%) who were re-examined due to the existing rib hump. The non-scoliotics were 1.5 - 2 years younger than the ones who had already developed scoliosis, and they both had a “rib index” of approximately 1.5. The DRCS was present in all referrals. In contrast, there was no scoliotic spine without it, as the DRCS was always present in scoliotic lateral spinal radiographs without exception. This observation supports the hypothesis.

A practical method of scoliosis detection using the Scoliometer as a multiple angle of trunk inclination system of back shape appraisal is reported by Burwell et al. [63]. We agree with the Discussant quoting Bunnell [59], that ribcage asymmetry does not always equate with vertebral column asymmetry.

#### Body mass index (BMI)

##### Comment no4

I cannot confirm the excess of TA associated with relatively lower Body Mass Index (BMI) in girls and boys. In my material there were children with scoliosis and with high body mass and also the children with lower body mass, more with lower BMI. It is not important for aetiology, only important for clinical examination. In a child with high BMI it is more difficult to see early clinical signs of scoliosis.

Response.

Our conclusions relating to trunk asymmetry and BMI are based on statistical analyses. Do you have statistical analyses on which to base your statements? If so, we can discuss your findings in relation to the aetiology of trunk asymmetry. (Moderator: Statistical findings have not been communicated by the Discussant).

#### BMI and later menarche

##### Comment no5

Dr. Grivas’ finding relating lower BMI to later menarche is established. Nature must ensure the human female has the ability to sustain a foetus so she will wait until a sufficient amount of fat is stored. According to Frisch [65], a minimum level of fatness (17% of body weight) is associated with menarche.

Response.

We agree! ‘Mother Nature’ must ensure the female has the ability to sustain a foetus so she will wait until a sufficient amount of fat is stored. We would also bear in mind that ‘Mother Nature’ is not creating on “credit” but on her own existing “wealth/energy” which in this case is fat. And it is very interesting to notice that the majority of scoliotics are in the group of low BMI.

The authors never claimed the ‘paternity’ of the statement relating lower BMI to later menarche, see [66-70].

##### Comment no6

Significantly later menarche in girls is found to be associated with more severe TAs. Later onset of menarche means that the period of growth is likely to be prolonged. In that case, it is difficult to explain why boys are less prone to severe TA given that their growth period is longer.

Response.

Girls mature earlier than boys. The onset of puberty in boys appears later than the age at menarche, a physiological event documented in human physiology. If menarche appears later, then the detrimental/aetiological factors responsible for scoliosis development in girls who are genetically susceptible to scoliosis have more time in their favour. In this connection, latitude is associated with age at menarche [66-69]; this explains why the prevalence of IS is more in northern latitudes than at the equator. This is useful knowledge in selecting the ages at which to screen girls for scoliosis (see response to Comment no. 11).

#### Biomechanical aetiology

##### Comment no7

My “biomechanical aetiology” [71], unchanged for 15 years, answers all questions connected with aetiology of the so-called idiopathic scoliosis

Response.

In your publications the lack of numerical recording of observations and quantitative data makes it very difficult to make a scientific appraisal of your “biomechanical aetiology”.

#### Posteriorly-directed shear loads on the pre-existing axially-rotated growing spine

##### Comment no8

Read with interest. My only suggestion is that the author considers Rene Castelein's observation that slight right thoracic vertebral rotation is normal in the adolescent population [29]. This seems to me to be a simpler and more direct explanation of right apex predominance than invoking hypothalamic functional asymmetry. It would still leave the rest of the hypothesis intact.

Response.

Professor Rene Castelein and colleagues reported that slight right thoracic axial vertebral rotation is a normal finding in the adult population [27]. This was confirmed in juvenile and adolescent children who were without evidence of spinal pathology [29,72]. In these children, no statistical significant difference for pre-existing vertebral rotation was found between the sexes. While these findings provide a direct explanation of right apex predominance of AIS, they do not account for:

•greater prevalence of girls than boys with RT-AIS

•sidedness of thoracolumbar and lumbar AIS, and

•larger severe TAs in girls

Also, there is evidence for a “central” (CNS)” and not a “local” (spinal) aetiology for truncal asymmetry [60]. A significant positive correlation between cerebral lateralization as expressed by handedness and mild trunk asymmetry at the mid-thoracic region suggests a pathogenic role for the cerebral cortex in determining thoracic surface morphology (see Comment 9 and response).

A developmental laterality seems to be preset in normal subjects [21,72] for thoracic ratio (TR) asymmetry (TRD) is evident below T2 and more in puberty than childhood. Table 8 in reference [21] shows that mean TR differences, statistically significant for asymmetry, are all negative, implying larger right than left thoracic ratios. This morphology was attributed to asymmetric muscular action, which is centrally directed.

There are sex differences of rib-vertebra angles (RVAs) during development reported for normal children [7]. The possible role of neuromuscular mechanisms being responsible for AIS pathogenesis are discussed by Grivas and colleagues [7,8].

#### Central nervous system (CNS)

##### Comment no9

I believe Dr. Grivas is correct that the primary force in the development of AIS is the CNS [73], with a critical role played by the hypothalamus. We await further results from research into the aetiology of AIS to identify the other brain structures which we suspect are involved.

Response.

There is increasing evidence and growing support for the possibility of an underlying neurological disorder in the aetiopathogenesis of AIS [1,2]. The evidence includes the spinal cord [74,75], hind brain [76], motor control [77], motor cortex [78], supplementary motor area [79], cerebral cortex [80] and vestibular system [58].

#### Sympathetic nervous system

##### Comment no10

Since the sympathetic nervous system extends on both sides of the spine, it is not difficult to explain why a hormonally-mediated mechanism should affect one side more than the other, and cause a right-sided predominance of truncal asymmetry.

Response.

Any sympathetic nervous system activation with asymmetry would lead to asymmetries in ribs and/or vertebrae producing trunk asymmetry when beyond the capacity of postural mechanisms of the somatic nervous system to control the shape distortion of the trunk.

#### Girls’ susceptibility to circulating leptin

##### Comment no11

I do not understand why girls should have increased susceptibility to circulating leptin. If there are boys and girls who have similar BMIs, why then are only girls more susceptible to leptins? In most endocrine disorders we see that both sexes are affected more or less similarly. Why should endocrine-imbalance-induced TA be an exception to this?

Response.

Healthy girls have higher serum leptin levels before, during, and after puberty than boys, even after accounting for the development of greater female adiposity [81]. The sexual dimorphism in leptin concentrations during puberty appears to be due also to a stimulatory effect of estradiol on fat deposition and leptin concentration in females, and a suppressive effect of testosterone on leptin concentration in males [81]. Hence, the bioavailability of leptin is different in healthy girls and boys [82,83].

We do not know why girls show more severe TAs than boys (see response to Comment no. 8). We suspect that this phenotypic sexual dimorphism of normal TAs is related to the onset and timing of puberty including 1) s*econdary sexual development, involving body composition*[2,84,85] with girls depositing more white adipose tissue that generates higher circulating leptin levels than boys; 2) *adolescent skeletal growth spurt*, and 3) a putative asymmetry *process* as suggested for AIS girls [11,12] (see response to Comment no. 2). Both 1) and 2) are initiated and controlled by hypothalamic mechanisms; The *asymmetry process* for which we discuss here autonomic nervous system mechanisms [11,13], does not exclude the possibility of somatic nervous system mechanisms contributing to the causation of TAs in some healthy subjects as suggested for the trunk deformity of some AIS girls [7,8,13].

## Appendix

In AIS subjects, asymmetries affecting the skeleton occur in the three cardinal planes – frontal, and particularly in the trunk, antero-posterior (sagittal, front-back), and transverse*. Bilateral (left-right or mirror image) symmetry* in animals describes a basic body plan in which left and right sides of the organism can be divided into approximate mirror images of each other along the midline [86]. *Bilateral asymmetry*, best known as *directional asymmetry*[87], involves one or other side of the body being of different sizes; in the 3-D deformity of AIS it is found in frontal and transverse planes, the latter as neurocentral physeal growth asymmetries. *Fluctuating asymmetry* (not considered here) - a particular form of bilateral asymmetry characterized by small random deviations from perfect bilateral symmetry, is commonly used as a measure of developmental instability from environmental and genetic origins [87,88].

## Competing interests

The authors declare that they have no competing interests.

## Authors’ contributions

Dr TBG devised the original questions to be put for discussion and wrote the responses. Professor GRB edited the responses and compiled the references for the responses. Dr PHD mailed the questions to the respondents and correspondents and had overall editorial control of the final paper. All authors read and approved the final manuscript.

## References

[B1] Wang WJ Yeung HY Chu WC Tang NL Lee KM Qiu Y Burwell RG Cheng JC Top theories for the etiopathogenesis of adolescent idiopathic scoliosis J Pediatr Orthop 2011 31 S14 S27 21173615 10.1097/BPO.0b013e3181f73c12

[B2] Burwell RG Dangerfield PH Whither the etiopathogenesis (and scoliogeny) of adolescent idiopathic scoliosis? Stud Health Technol Inform 2012 176 3 19 22744448

[B3] Stokes IA Burwell RG Dangerfield PH Biomechanical spinal growth modulation and progressive adolescent scoliosis – a test of the ‘vicious cycle’ pathogenetic hypothesis: summary of an electronic focus group debate of the IBSE Scoliosis 2006 1 16 10.1186/1748-7161-1-16 17049077 PMC1626075

[B4] Burwell RG Aujla RK Grevitt MP Randell TL Dangerfield PH Moulton A Anderson SI A new approach to the pathogenesis of adolescent idiopathic scoliosis: interaction between risk factors involving a diverse network of causal developmental pathways [Abstract] Clin Anat 2011 24 3 384

[B5] Goldberg CJ Moore DP Fogarty EE Dowling FE Scoliosis: a review Pediatr Surg Int 2008 24 129 144 10.1007/s00383-007-2016-5 17891405

[B6] Burwell RG Aujla RK Grevitt MP Dangerfield PH Moulton A Randell TL Pathogenesis of adolescent idiopathic scoliosis in girls. A double neuro-osseous theory involving disharmony between two nervous systems, somatic and autonomic expressed in the spine and trunk: possible dependency on sympathetic nervous system and hormones with implications for medical therapy Scoliosis 2009 4 24 10.1186/1748-7161-4-24 19878575 PMC2781798

[B7] Grivas T Burwell R Purdue M Webb J Moulton A Segmental patterns of rib-vertebra angles in chest radiographs of children: changes related to rib level, age, sex, side and significance for idiopathic scoliosis [Abstract] Clin Anat 1992 5 4 272 288 10.1002/ca.980050404

[B8] Burwell RG Cole AA Cook TA Grivas TB Kiel AW Moulton A Thirlwall AS Upadhyay SS Webb JK Wemyss-Holden SA Whitwell DJ Wojcik AS Wythers DJ Pathogenesis of idiopathic scoliosis. The Nottingham concept Acta Orthop Belg 1992 58 I 33 58 1456018

[B9] Grivas TB Burwell RG Mihas C Vasiliadis ES Triandaffylopoulos G Kaspiris A Relatively lower body mass index is associated with an excess of severe truncal asymmetry in healthy adolescents. Do body fat, leptin, hypothalamus and sympathetic nervous system influence truncal growth asymmetry Scoliosis 2009 4 1 13 10.1186/1748-7161-4-13 19566930 PMC2717060

[B10] Burwell RG Dangerfield PH Moulton A Grivas TB Adolescent idiopathic scoliosis (AIS), environment, exposome and epigenetics: a molecular perspective of postnatal normal spinal growth and the etiopathogenesis of AIS with consideration of a network approach and possible implications for medical therapy Scoliosis 2011 6 1 26 10.1186/1748-7161-6-26 22136338 PMC3293085

[B11] Burwell RG Aujla RK Grevitt MP Randell TL Dangerfield PH Cole AA Kirby AS Polak FJ Pratt RK Webb JK Moulton A Upper arm length model suggests a transient asymmetry process in the pathogenesis of right thoracic adolescent idiopathic scoliosis (RT-AIS) in girls [Abstract] Clin Anat 2011 24 3 384

[B12] Burwell RG Aujla RK Grevitt MP Randell TL Dangerfield PH Cole AA Kirby AS Polak FJ Pratt RK Webb JK Moulton A Upper arm length model suggests transient asymmetry is associated with right thoracic adolescent idiopathic scoliosis (RT-AIS) with implications for pathogenesis and estimation of linear skeletal overgrowth Stud Health Technol Inform 2012 176 188 194 22744488

[B13] Burwell RG Grevitt MP Randell TL Dangerfield PH Aujla RK Cole AA Pratt RK Webb JK Moulton A Anderson SI Abnormal bilateral skeletal asymmetries and their putative genetic and epigenetic origins in enantiomorphic growth plates of girls with adolescent idiopathic scoliosis (AIS) [Abstract] Clin Anat 2012 25 2 267

[B14] Nissinen M Heliovaara M Seitsamo J Poussa M Trunk asymmetry, posture, growth, and risk of scoliosis. A three-year follow-up of Finnish prepubertal school children Spine 1993 18 8 13 10.1097/00007632-199301000-00002 8434329

[B15] Hung VW Qin L Cheung CS Lam TP Ng BK Tse YK Go X Lee KM Cheng JC Osteopenia: a new prognostic factor of curve progression in adolescent idiopathic scoliosis J Bone Joint Surg Am 2005 87-A 2709 2716 16322621 10.2106/JBJS.D.02782

[B16] Mau H Zur Atiopathogenese von Scoliose, Huftdysplasie und Schiefhals im Sauglinsalter Zeitschrift f Orthop 1979 5 601 605 549336

[B17] Burwell RG James NJ Johnson F Webb JK Wilson YG Standardised trunk asymmetry scores, A study of back contour in healthy schoolchildren J Bone Joint Surg 1985 65.-B 4 1983 452–463 10.1302/0301-620X.65B4.6874719 6874719

[B18] Grivas TB Vasiliadis ES Mihas C Maziotou C Triantafyllopoulos G Alexandropoulos P Trunk asymmetry in normal juveniles Scoliosis 2009 4 Suppl 1 O7 10.1186/1748-7161-4-S1-O7

[B19] Grivas TB Samelis P Chadziargiropoulos T Polyzois B Study of the rib cage deformity in children with 10 degrees-20 degrees of Cobb angle late onset idiopathic scoliosis, using rib-vertebra angles – aetiologic implications Stud Health Technol Inform 2002 91 20 24 15457688

[B20] Burwell RG Aujla RK Freeman BJ Dangerfield PH Cole AA Kirby AS Polak FJ Pratt RK Moulton A The posterior skeletal thorax: rib-vertebral angle and axial vertebral rotation asymmetries in adolescent idiopathic scoliosis Stud Health Technol Inform 2008 140 263 268 18810034

[B21] Grivas TB Burwell RG Purdue M Webb JK Moulton A A segmental analysis of chest radiographs of children. Changes related to spinal level, age, sex, side and significance for lung growth and scoliosis J Anat 1991 178 21 38 1810928 PMC1260532

[B22] Grivas TB Dangas S Polyzois BD Samelis P The double rib contour sign (DRCS) in lateral spinal radiographs: aetiologic implications for scoliosis Stud Health Technol Inform 2002 88 38 43 15456003

[B23] Grivas TB Vasiliadis ES Mihas C Savvidou O The effect of growth on the correlation between the spinal and rib cage deformity: implications on idiopathic scoliosis pathogenesis Scoliosis 2007 14 2 11 17868459 10.1186/1748-7161-2-11 PMC2040132

[B24] Kiel AW Burwell RG Moulton A Purdue M Webb JK Wojcik AS Segmental patterns of sagittal spinal curvature in children screened for scoliosis: kyphotic angulation at the thoracolumbar region and the mortice joint Clin Anat 1992 8 353 371

[B25] Burwell RG Dangerfield PH Freeman BJ Concepts on the pathogenesis of adolescent idiopathic scoliosis. Bone growth and mass, vertebral column, spinal cord, brain, skull, extra-spinal left-right skeletal length asymmetries, disproportions and molecular pathogenesis Stud Health Technol Inform 2008 135 3 52 18401079

[B26] Grivas TB Dangas S Samelis P Maziotou C Kandris K Lateral spinal profile in school-screening referrals with and without late onset idiopathic scoliosis 10 degrees-20 degrees Stud Health Technol Inform 2002 91 25 31 15457689

[B27] Kouwenhoven JWM Vincken KL Bartels LW Castelein RM Analysis of pre-existent vertebral rotation in the normal spine Spine 2006 31 13 1467 1472 10.1097/01.brs.0000219938.14686.b3 16741456

[B28] Kouwenhoven JW Smit TH van der Veen AJ Kingma I van Dieën JH Castelein RM Effects of dorsal versus ventral shear loads on the rotational stability of the thoracic spine: a biomechanical porcine and human cadaveric study Spine 2007 32 23 2545 2550 10.1097/BRS.0b013e318158cd86 17978652

[B29] Janssen MM Kouwenhoven JW Schlösser TP Viergever MA Bartels LW Castelein RM Vincken KL Analysis of preexistent vertebral rotation in the normal infantile, juvenile, and adolescent spine Spine 2011 36 7 E486 E491 10.1097/BRS.0b013e3181f468cc 21240053

[B30] Kirby AS Burwell RG Cole AA Pratt RK Webb JK Moulton A Evaluation of a new real-time ultrasound method for measuring segmental rotation of vertebrae and ribs in scoliosis Stud Health Technol Inform 1999 59 316 320

[B31] Burwell RG Aujla RK Cole AA Kirby AS Pratt RK Webb JK Moulton A Sawatzky BJ Relation of rib deformity to vertebral deformity in the transverse plane at the curve apex in preoperative adolescent idiopathic scoliosis (AIS): an ultrasound, radiological and surface study of pathomechanisms International Research Society of Spinal Deformities Symposium 2004 2004 University of British Columbia 302 306

[B32] Normelli H Sevastik JA Ljung G Jönsson-Söderström AM The symmetry of the breasts in normal and scoliotic girls Spine 1986 11 7 749 752 10.1097/00007632-198609000-00016 3787348

[B33] Normelli H Sevastik J Walberg H The thermal emission from the skin and the vascularity of breasts in normal and scoliotic girls Spine 1985 11 5 405 408 3750076 10.1097/00007632-198606000-00001

[B34] Normelli H Sevastik J Akrivos J The length and ash weight of the ribs of normal and scoliotic persons Spine 1985 10 6 590 592 10.1097/00007632-198507000-00015 4081873

[B35] Sevastik JA The thoracospinal concept of the etiopathogenesis of idiopathic scoliosis, Etiology of Adolescent Idiopathic Scoliosis: current trends and relevance to new treatment approaches State of the Art Reviews: Spine 2000 14 2 391 400

[B36] Sevastik JA Dysfunction of the autonomic nerve system (ANS) in the aetiopathogenesis of adolescent idiopathic scoliosis Stud Health Technol Inform 2002 88 20 23 15455999

[B37] Sevastik J Burwell RG Dangerfield PH A new concept for the etiopathogenesis of the thoracospinal deformity of idiopathic scoliosis: summary of an electronic focus group debate of the IBSE Eur Spine J 2003 12 440 450 10.1007/s00586-002-0489-4 12955611 PMC3467792

[B38] Qiu Y Sun GQ Zhu F Wang WJ Zhu ZZ Rib length discrepancy in patients with adolescent idiopathic scoliosis Stud Health Technol Inform 2010 158 63 66 20543401

[B39] Zhu F Chu WC Sun G Zhu ZZ Wang WJ Cheng JC Qiu Y Rib length asymmetry in thoracic adolescent idiopathic scoliosis: is it primary or secondary? Eur Spine J 2011 20 2 254 259 10.1007/s00586-010-1637-x 21120673 PMC3030715

[B40] Iliopoulos P Korovessis P Koureas G Zacharatos S Stergiou P Asymmetric evolution of anterior chest wall blood supply in female adolescents with progressive right-convex thoracic idiopathic scoliosis Eur Spine J 2007 16 1343 1346 10.1007/s00586-007-0322-1 17294053 PMC2200743

[B41] Korovessis P Iliopoulos P Koureas G Zacharatos S Stergiou P Evolution of anterior chest wall blood supply in female adolescents with progressive right-convex thoracic idiopathic scoliosis J Spinal Disord Tech 2007 20 3 190 194 10.1097/01.bsd.0000211267.66615.ac 17473637

[B42] Schwender JD Denis F Coronal plane imbalance in adolescent idiopathic scoliosis with left lumbar curves exceeding 40° Spine 2000 25 18 2358 2363 10.1097/00007632-200009150-00015 10984789

[B43] Burwell RG Aujla RK Freeman BJC Dangerfield PH Cole AA Kirby AS Pratt RK Webb JK Moulton A Patterns of extra-spinal left-right skeletal asymmetries in adolescent girls with lower spine scoliosis: relative lengthening of the ilium on the curve concavity and of right lower limb segments Stud Health Technol Inform 2006 123 57 65 17108404

[B44] Burwell RG Freeman BJC Dangerfield PH Aujla RK Cole AA Kirby AS Pratt RK Webb JK Moulton A Left-right upper arm length asymmetry associated with apical vertebral rotation in subjects with thoracic scoliosis: anomaly of bilateral symmetry affecting vertebral, costal and upper arm physes? Stud Health Technol Inform 2006 123 66 71 and *J Bone Joint Surg Br* 2008 **90-B:**Supp III, 476 17108405

[B45] Burwell RG Aujla RK Grevitt MP Randell TL Dangerfield PH Cole AA Kirby AS Polak FJ Pratt RKM Webb JK Moulton A Right thoracic adolescent idiopathic scoliosis (RT-AIS) in girls: abnormal bilateral asymmetry of upper limb lengths involves forearm-with-hands as well as upper arms [Abstract] Clin Anat 2011 24 4 530

[B46] Saji MJ Upadhyay SS Leong JC Increased femoral neck shaft angles in adolescent idiopathic scoliosis Spine 1995 20 3 303 311 10.1097/00007632-199502000-00008 7732466

[B47] Burwell RG Aujla RK Kirby AS Freeman BJC Cole AA Dangerfield PH Polak FJ Pratt RK Webb JK Moulton A Ultrasound femoral anteversion (FAV) is decreased and asymmetric after school screening for adolescent idiopathic scoliosis (AIS): femora show torsional anomalies that if in the trunk may initiate the deformity [Abstract] Clin Anat 2007 20 7 855

[B48] Burwell RG Aujla RK Kirby AS Moulton A Dangerfield PH Freeman BJC Cole AA Polak FJ Pratt RK Webb JK Ultrsound femoral anteversion (FAV) and tibial torsion (TT) after school screening for adolescent idiopathic scoliosis (AIS) Stud Health Technol Inform 2008 140 225 230 18810028

[B49] Burwell RG Aujla RK Kirby AS Freeman BJC Cole AA Dangerfield PH Polak FJ Pratt RK Webb JK Moulton A Ultrasound femoral anteversion/tibial torsion correlations are significant, abnormal and asymmetric after school screening for adolescent scoliosis (AIS): lower limb torsional markers for initiation of the torsional trunk deformity of AIS? [Abstract] Clin Anat 2007 20 7 855

[B50] Burwell RG Aujla RK Kirby AS Moulton A Dangerfield PH Freeman BJC Cole AA Polak FJ Pratt RK Webb JK Ultrasound femoral anteversion (FAV) relative to tibial torsion (TT) is abnormal after school screening for adolescent idiopathic scoliosis (AIS): evaluation by two methods Stud Health Technol Inform 2008 140 37 43 18809996

[B51] Burwell RG Aujla RK Freeman BJ Dangerfield PH Cole AA Kirby AS Pratt RK Webb JK Moulton A Patterns of extra-spinal left-right skeletal asymmetries and proximo-distal disproportion in adolescent girls with lower spine scoliosis: ilio-femoral length asymmetry & bilateral tibial/foot length disproportion Stud Health Technol Inform 2006 123 101 108 17108411

[B52] Burwell RG Aujla RK Freeman BJC Cole AA Dangerfield PH Kirby AS Pratt RK Webb JK Moulton A Proximo-distal skeletal length disproportion in lower limbs of girls with adolescent idiopathic scoliosis compared with normal girls: tibial length/foot length is greater bilaterally and associated with left-right tibial length asymmetry [Abstract] Clin Anat 2007 20 4 467

[B53] Burwell RG Aujla RK Kirby AS Freeman BJC Cole AA Dangerfield PH Polak FJ Pratt RK Webb JK Moulton A Ultrasound tibial torsion (TT) and TT asymmetry are not abnormal after school screening for adolescent idiopathic scoliosis (AIS): in scoliosis boys TT is decreased relative to scoliosis girls without asymmetry - the result of altered maturation at knee tibial growth plates? [Abstract] Clin Anat 2007 20 7 855

[B54] Shi L Heng PA Wong TT Chu WC Yeung BH Cheng JC Morphometric analysis for pathological abnormality detection in the skull vaults of adolescent idiopathic scoliosis girls Med Image Comput Comput Assist Interv 2006 9 Pt 1 175 182 17354888 10.1007/11866565_22

[B55] Mehta MH Moreland MS, Pope MH, Armstrong GWD Moiré topography and associated asymmetries in scoliosis Moiré topography and spinal deformities 1981 New York: Pergamon Press 186 189

[B56] Pecina M Lulic-Ducik O Pecina-Hrncevic A Hereditary orthodontic anomalies and idiopathic scoliosis Int.Orth (SICOT) 1991 15 57 59 10.1007/BF00210536 2071283

[B57] Ben-Bassat Y Yitschaky M Kaplan L Brin I Occlusal patterns in patients with idiopathic scoliosis Am J Orthod Dentofacial Orthop 2006 130 5 629 633 10.1016/j.ajodo.2005.01.032 17110260

[B58] Shi L Wang D Chu WC Burwell GR Wong TT Heng PA Cheng JC Automatic MRI segmentation and morphoanatomy analysis of the vestibular system in adolescent idiopathic scoliosis NeuroImage 2011 54 Suppl 1 S180 S188 20382235 10.1016/j.neuroimage.2010.04.002

[B59] Bunnell WP An objective criterion for scoliosis screening J Bone Joint Surg Am 1984 66 9 1381 1387 6501335

[B60] Grivas TB Vasiliadis ES Polyzois VD Mouzakis V Trunk asymmetry and handedness in 8245 school children Pediatr Rehabil 2006 9 3 259 266 10.1080/10428190500343027 17050403

[B61] Murrell GA Coonrad RW Moorman CT III Fitch RD An assessment of the reliability of the scoliometer Spine 1993 18 709 712 10.1097/00007632-199305000-00006 8516699

[B62] Huang SC Effectiveness of scoliometer in school screening for scoliosis J Formosan Med Assoc (Taiwan I Hsueh Hui Tsa Chih) 1988 87 955 959 3241154

[B63] Burwell RG Patterson JF Webb JK Wojcik AS Neugebauer H, Windischbauer G School screening for scoliosis – The multiple ATI system of back shape appraisal using the Scoliometer with observations on the sagittal declive angle Surface topography and body deformity. Proceedings of the 5th International Symposium September 29-October 1, 1988 Wien 1990 New York: Gustav Fischer Verlag 17 23

[B64] Amendt LE Ause-Ellias KL Eybers JL Wadsworth CT Nielsen DH Weinstein SL Validity and reliability testing of the scoliometer Phys Ther 1990 70 108 117 2296610 10.1093/ptj/70.2.108

[B65] Frisch RE Body fat, menarche, fitness and fertility Hum Reprod 1987 2 6 521 533 3117838 10.1093/oxfordjournals.humrep.a136582

[B66] Grivas TB Mihas C Lepoura N Maheriotis C Dascalakis P Mariolis A The role of geographic latitude to the age at menarche. Poster No 1008, World Organization of National Colleges, Academies and Academic Associations of General Practitioners/Family Physicians11th Conference of the European Society of General Practice/Family Medicine, Kos Island, Greece, September 3–7 2005. In: Proceedings of the WONCA Europe 2005 50 51

[B67] Grivas TB Mouzakis V Vasiliadis E Mihas K Polyzois V Why the prevalence of AIS is different in various countries? Relation to geographic latitude and the possible role of the age at menarche. 12^th^ IMAST, Fairmont Banff, Alberta, Canada, July 7–9, 2005 Proceedings of the 12th IMAST Abstract No. 48, 40th SRS Annual Meeting & Course, Loews Miami Beach Hotel Miami, Florida USA, October 27–30, 2005. In Proceedings of the 40th SRS Annual Meeting & Course 2005

[B68] Grivas TB Vasiliadis E Savvidou O Mouzakis V Koufopoulos G Geographic latitude and prevalence of adolescent idiopathic scoliosis Stud Health Technol Inform 2006 123 84 89 17108408

[B69] Grivas T Vasiliadis E Mouzakis V Mihas C Koufopoulos G Association between adolescent idiopathic scoliosis prevalence and age at menarche in different geographic latitudes Scoliosis 2006 1 9 10.1186/1748-7161-1-9 16759371 PMC1501058

[B70] Mao SH Jiang J Sun X Zhao Q Qian BP Liu Z Shu H Qiu Y Timing of menarche in Chinese girls with and without adolescent idiopathic scoliosis: current results and review of the literature Eur Spine J 2011 20 2 260 265 10.1007/s00586-010-1649-6 21153847 PMC3030718

[B71] Karski T Explanation of biomechanical etiology of the so-called idiopathic scoliosis (1995–2007). New clinical and radiological classification Locomotor System 2010 17 1–2 26 42

[B72] Castelein RM Pre-existent rotation of the normal spine at different ages and its consequences for the scoliotic mechanism Stud Health Technol Inform 2012 176 20 25 22744449

[B73] McMaster ME Heated indoor swimming pools, infants, and the pathogenesis of adolescent idiopathic scoliosis: a neurogenic hypothesis Environ Health 2011 10 1 86 10.1186/1476-069X-10-86 21975145 PMC3213202

[B74] Chu WC Lam WW Chan YL Ng BK Lam TP Lee KM Guo X Cheng JC Relative shortening and functional tethering of spinal cord in adolescent idiopathic scoliosis? Study with multiplanar reformat magnetic resonance imaging and somatosensory evoked potential Spine 2006 31 E19 E25 10.1097/01.brs.0000193892.20764.51 16395162

[B75] Chu WCW Lam WWM Ng BKW Tze-Ping L Lee KM Guo X Cheng JC Burwell RG Dangerfield PH Jaspan T Relative shortening and functional tethering of spinal cord in adolescent scoliosis—result of asynchronous neuro-osseous growth? Summary of an electronic focus group debate of the IBSE Scoliosis 2008 3 8 10.1186/1748-7161-3-8 18588673 PMC2474583

[B76] Lowe TG Edgar M Margulies JY Miller NH Raso VJ Reinker KA Rivard CH Current concepts review: etiology of idiopathic scoliosis: current trends in research J Bone Joint Surg 2000 82-A 1157 1168 10954107 10.2106/00004623-200008000-00014

[B77] Herman R Mixon J Fisher A Maulucci R Stuyck J Idiopathic scoliosis and the central nervous system: a motor control problem. The Harrington Lecture, 1983. Scoliosis Research Society Spine 1985 10 1 14 10.1097/00007632-198501000-00001 3885413

[B78] Doménech J Tormos JM Barrios C Pascual-Leone A Motor cortical hyperexcitability in idiopathic scoliosis: could focal dystonia be a subclinical etiological factor? Eur Spine J 2010 19 223 230 10.1007/s00586-009-1243-y 20033462 PMC2899814

[B79] Domenech J García-Martí G Martí-Bonmatí L Barrios C Tormos JM Pascual-Leone A Abnormal activation of the motor cortical network in idiopathic scoliosis demonstrated by functional MRI Eur Spine J 2011 20 7 1069 1078 10.1007/s00586-011-1776-8 21499781 PMC3176702

[B80] Wang D Shi L Chu WC Burwell RG Cheng JC Ahuja AT Abnormal cerebral cortical thinning pattern in adolescent girls with idiopathic scoliosis NeuroImage 2012 59 2 935 942 10.1016/j.neuroimage.2011.07.097 21872666

[B81] Demerath EW Towne B Wisemandle W Blangero J Chumlea WC Siervogel RM Serum leptin concentration, body composition, and gonadal hormones during puberty Int J Obes Relat Metab Disord 1999 23 7 678 685 10.1038/sj.ijo.0800902 10454100

[B82] Liu Z Tam EM Sun GQ Lam TP Zhu ZZ Sun X Lee KM Ng TB Qiu Y Cheng JC Yeung HY Abnormal leptin bioavailability in adolescent idiopathic scoliosis - an important new finding Spine 2012 37 7 599 604 10.1097/BRS.0b013e318227dd0c 21681139

[B83] Lombardi G Akoume MY Colombini A Moreau A Banfi G Biochemistry of adolescent idiopathic scoliosis Adv Clin Chem 2011 54 165 182 21874761 10.1016/b978-0-12-387025-4.00007-8

[B84] Davey RC Cochrane T Dangerfield PH Chockalingam N Dorgan JC Sawatzky BJ Anthropometry and body composition in females with adolescent idiopathic scoliosis International Research Society of Spinal Deformities Symposium 2004 2004 University of British Columbia 323 326

[B85] Barrios C Cortés S Pérez-Encinas C Escrivá MD Benet I Burgos J Hevia E Pizá G Domenech P Anthropometry and body composition profile of girls with nonsurgically treated adolescent idiopathic scoliosis Spine 2011 36 18 1470 1477 10.1097/BRS.0b013e3181f55083 21242873

[B86] Burwell RG Dangerfield PH Freeman BJ Aujla RK Cole AA Kirby AS Pratt RK Webb JK Moulton A Etiologic theories of idiopathic scoliosis: the breaking of bilateral symmetry in relation to left-right asymmetry of internal organs, right thoracic adolescent idiopathic scoliosis (AIS) and vertebrate evolution Stud Health Technol Inform 2006 123 385 390 17108456

[B87] Graham JH Antisymmetry, directional asymmetry, and dynamic morphogenesis Genetica 1993 89 1–3 121 137

[B88] Dangerfield PH Scutt D Ashton S Manning JT Dorgan JC Idiopathic scoliosis and fluctuating asymmetry (FA) Stud Health Technol Inform 1997 37 45 48

